# Effects of computerized cognitive training on structure‒function coupling and topology of multiple brain networks in people with mild cognitive impairment: a randomized controlled trial

**DOI:** 10.1186/s13195-023-01292-9

**Published:** 2023-09-23

**Authors:** Jingsong Wu, Youze He, Shengxiang Liang, Zhizhen Liu, Jia Huang, Weilin Liu, Jing Tao, Lidian Chen, Chetwyn C. H. Chan, Tatia M. C. Lee

**Affiliations:** 1 College of Rehabilitation Medicine, Fujian University of Traditional Chinese Medicine, Fuzhou, China; 2 The Academy of Rehabilitation Industry, Fujian University of Traditional Chinese Medicine, Fuzhou, China; 3 National-Local Joint Engineering Research Center of Rehabilitation Medicine Technology, Fujian University of Traditional Chinese Medicine, Fuzhou, China; 4 Fujian Key Laboratory of Rehabilitation Technology, Fujian University of Traditional Chinese Medicine, No. 1 Huatuo Road Shangjie Minhou, Fuzhou, China; 5 Department of Psychology, The Education University of Hong Kong, Tai Po, Hong Kong, China; 6 State Key Laboratory of Brain and Cognitive Sciences, The University of Hong Kong, Pokfulam Road, Hong Kong, China; 7 Laboratory of Neuropsychology and Human Neuroscience, The University of Hong Kong, Pokfulam Road, Hong Kong, China

**Keywords:** Mild cognitive impairment, Computerized cognitive training, Structural‒functional coupling, Topological attribute, Cognition

## Abstract

**Background:**

People with mild cognitive impairment (MCI) experience a loss of cognitive functions, whose mechanism is characterized by aberrant structure‒function (SC-FC) coupling and topological attributes of multiple networks. This study aimed to reveal the network-level SC-FC coupling and internal topological changes triggered by computerized cognitive training (CCT) to explain the therapeutic effects of this training in individuals with MCI.

**Methods:**

In this randomized block experiment, we recruited 60 MCI individuals and randomly divided them into an 8-week multidomain CCT group and a health education control group. The neuropsychological outcome measures were the Montreal Cognitive Assessment (MoCA), Chinese Auditory Verbal Learning Test (CAVLT), Chinese Stroop Color–Word Test (SCWT), and Rey–Osterrieth Complex Figure Test (Rey CFT). The brain imaging outcome measures were SC-FC coupling and topological attributes using functional MRI and diffusion tensor imaging methods. We applied linear model analysis to assess the differences in the outcome measures and identify the correspondence between the changes in the brain networks and cognitive functions before and after the CCT.

**Results:**

Fifty participants were included in the analyses after the exclusion of three dropouts and seven participants with low-quality MRI scans. Significant group × time effects were found on the changes in the MoCA, CAVLT, and Rey CFT recall scores. The changes in the SC-FC coupling values of the default mode network (DMN) and somatomotor network (SOM) were higher in the CCT group than in the control group (*P*(unc.) = 0.033, *P*(unc.) = 0.019), but opposite effects were found on the coupling values of the visual network (VIS) (*P*(unc.) = 0.039). Increasing clustering coefficients in the functional DMN and SOM and subtle changes in the nodal degree centrality and nodal efficiency of the right dorsal medial prefrontal cortex, posterior cingulate cortex, left parietal lobe, somatomotor area, and visual cortex were observed in the CCT group (*P* < 0.05, Bonferroni correction). Significant correspondences were found between global cognitive function and DMN coupling values (*P*(unc.) = 0.007), between immediate memory and SOM as well as FPC coupling values (*P*(unc.) = 0.037, *P*(unc.) = 0.030), between delayed memory and SOM coupling values (*P*(unc.) = 0.030), and between visual memory and VIS coupling values (*P*(unc.) = 0.007).

**Conclusions:**

Eight weeks of CCT effectively improved global cognitive and memory functions; these changes were correlated with increases in SC-FC coupling and changes in the topography of the DMN and SOM in individuals with MCI. The CCT regimen also modulated the clustering coefficient and the capacity for information transformation in functional networks; these effects appeared to underlie the cognitive improvement associated with CCT.

**Trial registration:**

Chinese Clinical Trial Registry, ChiCTR2000034012. Registered on 21 June 2020.

**Supplementary Information:**

The online version contains supplementary material available at 10.1186/s13195-023-01292-9.

## Introduction

Mild cognitive impairment (MCI) is associated with a progressive loss of memory and other neurocognitive functions, including attention, visuospatial abilities, and executive functions [1]. The conversion rate from MCI to dementia is approximately 10% per year [2]. Previous studies have reported abnormal structural and functional changes in multiple brain networks in people with MCI. These changes included reduced neural activities and connectivity involving the default mode, sensorimotor, and central executive networks [3–5]. Structural‒functional (SC-FC) coupling measures correlations between structural and functional connections, reflecting the between-network consistency, which could be markers to detect subtle pathological abnormalities with low consistency in SC-FC coupling [6–8]. As cognitive functions are mostly subserved by a network rather than a single neural substrate, SC-FC coupling has been found to yield stronger correlations with cognitive abilities such as memory and reasoning than a single neural substrate [9]. In addition, previous studies reported that coupling was more sensitive than the single model in detecting pathological abnormalities [8–10]. It has also been proven to be related to cognitive functions in individuals with MCI [9, 11]. Graph theory analysis reveals the intrinsic network properties (i.e., efficiency, degree centrality, and the small world) within a structural or functional network [6, 11]. Topological abnormalities within a network have been reported to be related to cognitive deficits in individuals with MCI, such as decreased network efficiency and clustering coefficients [12–14]. Abnormal topological properties of the SC and FC networks may help explain the changes in SC-FC coupling values.

Computerized cognitive training (CCT) has been demonstrated to be a safe and effective means of improving neurocognitive functions [15]. A few meta-analytic studies reported that CCT improved cognitive functions in MCI patients associated with structural or functional changes in specific brain regions [16–19]. Regarding structural changes, for example, Zhang et al. found that two sessions of CCT per week for 12 weeks increased gray matter volume in the right angular gyrus of MCI patients, which was associated with improved immediate memory function [20]. Na et al. showed that 24 sessions of CCT increased the cortical thickness and anisotropy values of the anterior cingulate gyrus in MCI patients [21]. Regarding functional changes, Tang et al. demonstrated that a 7-week CCT regimen enhanced FC between the left dorsolateral prefrontal cortex and medial prefrontal cortex in MCI patients [22]. Li et al. reported that 6 months of CCT significantly increased the amplitude of low-frequency fluctuation of BOLD signals at the bilateral temporal poles, insular cortices, and hippocampi in MCI patients [23]. However, each of these studies adopted a single-modality approach, i.e., structural or functional, which would have limited the interpretation of the brain-to-performance correspondence. The structural or functional approach alone would inevitably undervalue the treatment effects of CCT compared to a multimodal approach.

This study adopted SC-FC coupling and network-level topography to elucidate the treatment effects of an 8-week CCT regimen using a randomized controlled trial method. Positive CCT results were reported in a recently published paper [24]. In addition, the association between the positive clinical effects and the resting-state functional connectivity subserving episodic memory in the Papez circuit was illustrated in the same paper. We hypothesized that CCT would increase SC-FC network coupling and improve the topological attributes of the networks. The augmented coupling and topological attributes of the structural and functional networks would be associated with positive changes in the neurocognitive functions of the participants. We also hypothesized that CCT-related network changes would be specific to the cognitive improvements by CCT. Our findings can offer new insight into the potential neural mechanisms underpinning the therapeutic effects of CCT on cognitive function in MCI patients.

## Methods

### Study design and participants

This clinical trial of CCT was a multicenter, single-blind randomized controlled trial for MCI patients. In this paper, we applied a data-driven approach to explore the changes in SC-FC coupling and topological attributes using diffusion tensor imaging (DTI) and resting-state imaging. The inclusion criteria for the participants were as follows: (1) they met the diagnostic criteria for MCI; (2) they were 50 to 85 years old; (3) they had Montreal Cognitive Assessment (MoCA) scores of ≤ 25 points (1 point lower, i.e., ≤ 24 points, if they had < 12 years of education [25]); (4) they were in stage two (very mild cognitive decline) or three (mild cognitive decline) on the Global Deterioration Scale (GDS) [26]; (5) they were willing to join the study and sign the informed consent form; (6) they were right-handed; and (7) they had no MRI contraindications such as a cardiac pacemaker, metal implants, fixed dentures, or a high fever. The exclusion criteria were as follows: (1) uncontrolled hypertension (systolic blood pressure over 160 mmHg or diastolic blood pressure over 100 mmHg after taking medicine); (2) a diagnosis of dementia or any other psychiatric disease according to the fifth edition of the Diagnostic and Statistical Manual of Mental Disorders (DSM-V) [27]; (3) a history of alcohol or drug abuse; (4) a history of psychiatric disorders; (5) diseases of other body systems that might cause noncooperation with the intervention and evaluation, including severe organ failure, myocardial infarction or musculoskeletal diseases; (6) > 10 score on the Hamilton Rating Scale for Depression (HAMD) [28]; (7) ≥ 5 score on the Hachinski Ischemic Scale (HIS) [29]; (8) intake, in the last 2 weeks, of tranquilizers, antidepressants, psychostimulants or any other drugs that might affect cognitive function; (9) participation in other studies that might affect the result of the study; and (10) inability to undergo an MRI scan.

All potential participants were recruited from the clinics of the Affiliated Rehabilitation Hospital, Affiliated People’s Hospital, and Affiliated Second People’s Hospital of Fujian Traditional Chinese Medicine University. Diagnosis of the participants was made by two independent clinical neurologists according to Petersen’s criteria for MCI [30]. Screening of the potential participants was conducted by a research clinician in each hospital according to the inclusion and exclusion criteria. Using a computer-generated sequence, a research team member not involved in the intervention or assessment randomized the participants into the CCT and control groups in blocks of four. Pairs of rehabilitation therapists in each participating hospital received training on the interventions: one for the CCT group and the other for the control group. They delivered the corresponding interventions to the participants according to the study protocols set by the research team. Another rehabilitation therapist from each hospital received training on the administration of the clinical measures at baseline and after the participants completed the CCT regimen.

### Intervention

The CCT group received 24 1-h sessions (three sessions/week × 8 weeks) using the Chinese Food and Drug Administration (FDA)-approved Cognitive Assessment and Rehabilitation Training Machine (hereinafter, the Machine; Xiamen Amity Brain Health, China, No. YJRZ-LJ-01). The machine delivers clinical evaluation and training packages in electronic format for the rehabilitation of patients with neurological disorders. The CCT utilized the machine’s eleven-module cognitive training battery. We have reported positive treatment effects of the same 24-session CCT elsewhere [24].

The clinical protocol for the CCT spanned 2 weeks of inpatient treatment followed by 6 weeks of outpatient visits to the rehabilitation departments of the participating hospitals. In each session, the participants sat in front of computer screens and completed the training modules assigned by the rehabilitation therapist. Descriptions and assignment logistics of the eleven training modules, such as attention, working memory, and response speed, can be found in Supplementary Table S1. In general, the participants would first be assigned the training tasks corresponding to the deficits reflected by MoCA test items at the time of screening. Then, the participants were assigned other training tasks in the cognitive training battery in numerical order (from #1 to #11). Within a task, the participants began with the first difficulty level (easiest) and then were upgraded to a higher difficulty level in the next session when accuracy rates reached 80% or above. The same sequence of tasks continued throughout the 8 weeks.

Participants in the control group received eight 1-h weekly sessions of health education through outpatient visits to the hospital rehabilitation departments. The contents covered in the sessions were collated based on the Alzheimer’s disease prevention guidelines published by Barnard et al. [31]. The contents included various risk factors for cognitive impairment or dementia and prevention strategies based on dietary and lifestyle measures. All sessions were conducted by two rehabilitation therapists who received training on the contents and the one-on-one delivery method. The participants in the control group were instructed to refrain from engaging in any cognitive training.

### Outcome measures — neuropsychological tests

The tests below were administered to the participants on two occasions: at baseline and within 1 week after completion of the 8-week interventions.

#### Primary outcome

##### General neurocognitive status

The Fuzhou version of the Chinese MoCA (MoCA-ChiFZ) measures global cognitive function covering eight domains [32]. The MoCA-ChiFZ showed satisfactory Cronbach’s α and test–retest reliability (1-week delay) values of 0.92 and 0.90, respectively.

#### Secondary outcomes

##### Processing speed

The Digit Symbol Substitution Test (DSST) assesses the speed at which the participant can match a series of symbols to digits (from one to nine) [33]. Performance was quantified as the number of correct matches in 90 s.

##### Cognitive flexibility

The Chinese version of the Stroop Color–Word Test assesses cognitive flexibility in terms of the ability to inhibit an overlearned response [34]. Performance was measured using the accuracy and time taken to name the colors of the dots and words.

##### Episodic memory

The Chinese Auditory Verbal Learning Test (CAVLT) assesses episodic memory in terms of immediate recall, delayed recall, and delayed recognition [35]. Performance was quantified as the number of learned words recalled at different times during the test out of 15 learned words.

##### Nonverbal memory

The Chinese version of the Rey–Osterrieth Complex Figure Test (Rey CFT) – Delayed Recall was used to tap into the nonverbal memory function of the participants [36]. Performance was quantified as the number of learned figures recalled after a delay period.

### Image acquisition and data preprocessing

Each participant completed two MRI scans: one at baseline and one within a week after completing the intervention. Both the baseline and postintervention scans were performed with a Siemens Prisma 3.0-Tesla system (Erlangen, Germany) located at one participating hospital. Scans included T1-weighted imaging, DTI, and resting-state functional MRI (fMRI). T1 images were obtained using a magnetization-prepared rapid gradient-echo (MPRAGE) T1-weighted sequence with the following parameters: 256 × 256 matrix size with 192 contiguous slices, 1 mm isotropic resolution, repetition time (TR) = 2530 ms, echo time (TE) = 2.51 ms, flip angle (FA) = 7°, field of view (FOV) = 256 × 256 mm^2^, voxel size = 1.0 × 1.0 × 1.0 mm^3^. The DTI scans were acquired using a single-shot echo-planar imaging sequence with the following parameters: 64 diffusion directions with *b* = 1000 s/mm^2^, FOV = 224 mm × 224 mm^2^, TR = 8400 ms, TE = 64.0 ms, slice thickness = 2.0 mm, slice = 75, and voxel size = 2.0 × 2.0 × 2.0 mm^3^. The parameters of the resting-state fMRI sequence were as follows: FOV = 224 mm × 224 mm^2^, voxel size = 3.5 × 3.5 × 3.5 mm^3^, 37 contiguous slices of 2 mm thickness, TR = 2000 ms, TE = 30.0 ms, and FA = 90 degrees.

Preprocessing of DTI data was conducted following the steps of the PANDA (http://www.nitrc.org/projects/panda/) toolbox [37]. For the DTI data, the fractional anisotropy (FA) in a deterministic fiber network was generated for the fiber tract connectivity. The tractography was terminated if the turn angle between two consecutive directions of movement was greater than 45°or if the FA value of any voxel was out of the threshold range of 0.2–1.0. Preprocessing of resting-state fMRI data was based on the DPABI toolbox protocol (http://rfmri.org/dpabi) [38]. Spatial smoothing was performed using a Gaussian filter with a 6 mm full width at half maximum. Next, covariates (such as the signals of white matter and cerebrospinal fluid) were regressed out, the data were detrended, and a bandpass temporal filtering of 0.1 to 1.0 Hz was applied. The images were excluded if the head motion exceeded 3 mm of three-dimensional translation or 3 degrees of three-dimensional rotation.

### Network construction

The structural and functional networks composed of 300 regions of interest (ROIs) in the Schaefer-300 template [39] and the workflow of the network construction are summarized in Supplementary Fig. S1. To examine the network-level changes in SC-FC coupling, we applied the canonical seven-network parcellation defined by Yeo et al. [40], which comprises the default mode network (DMN), frontoparietal control network (PFC), dorsal attention network (DOR), ventral attention network (VEN), somatomotor network (SOM), visual network (VIS), and limbic network (LIM). Details of constructing the structural–functional connectivity and coupling are presented in the Supplementary Materials.

### Graph theory analysis

Participants’ topological attributes were computed using the GRETNA Toolbox [41]. Key topological graphic indices were calculated within the sparsity range of 5–50% (step 1%) [42], including network nodal degree centrality and nodal efficiency. Nodal efficiency is the capacity of a node for information transformation with other nodes in the network. Nodal degree centrality is a statistical characteristic determined from the connections between nodes by counting the number of direct connections to a given node, representing the importance of the node in the network [6, 43].

### Statistical analysis

All statistical analyses were performed in SPSS version 24.0 by an independent statistician who was blinded to group allocations. Between-group differences in the demographic characteristics were compared. The Group × Timepoint interaction effect on the outcome measures was tested with a linear mixed model (LMM) for repeated measures. In addition, minimal clinically important differences (MCIDs) were computed for the CCT and control groups. The MCID threshold was set at 0.5 standard deviations [44]. In other words, the smallest worthwhile change in scores as a result of the computer training program was defined as an increase of 0.5 standard deviations. Then, participants who showed improvements above the MCID threshold were selected and matched with those in the control group using the propensity score matching method, with age, gender, and years of education as covariates.

Between-group differences in the mean changes in the secondary measures, i.e., SC-FC coupling, and network-level topological properties were tested with a generalized linear model (GLM) while controlling for age, gender, and years of education. The tests for the main effects and multiple comparisons were two-tailed, and statistical significance was set at *P* < 0.05. No correction for multiple comparisons was adopted, as the brain networks under study subserve relatively different brain functions, and familywise error may not be applicable. Pearson’s correlation coefficient was calculated between the nonzero edges of the structural connectivity network and the corresponding elements of the functional connectivity matrix to measure the coupling values of each individual. After computing the mean changes, we used a GLM to compare the coupling values across groups while controlling for age, gender, and years of education. In the nodal topology comparison, GRETNA software was applied to conduct a two-sample *t* test with Bonferroni correction. Finally, we conducted partial correlation analyses to examine the relationships between the participants’ changes in the scores on the cognitive tests and the altered MRI outcomes after controlling for age, gender, and years of education with a threshold of *P* < 0.05, uncorrected (*P*(unc.)). The uncorrected* P* < 0.05 was set with reference to that adopted in previous similar studies [20, 22, 45]. Corrected *P* levels, such as Bonferroni adjustment (*P* < 0.05/76 = 0.0006), were not considered here because of the explorative nature of the analyses. The stringent corrected *P* threshold would lower the meaningfulness of the findings, particularly between the multimodal nature results of the participants’ neurobehavioral and MRI changes.

## Results

**Fig. 1:**
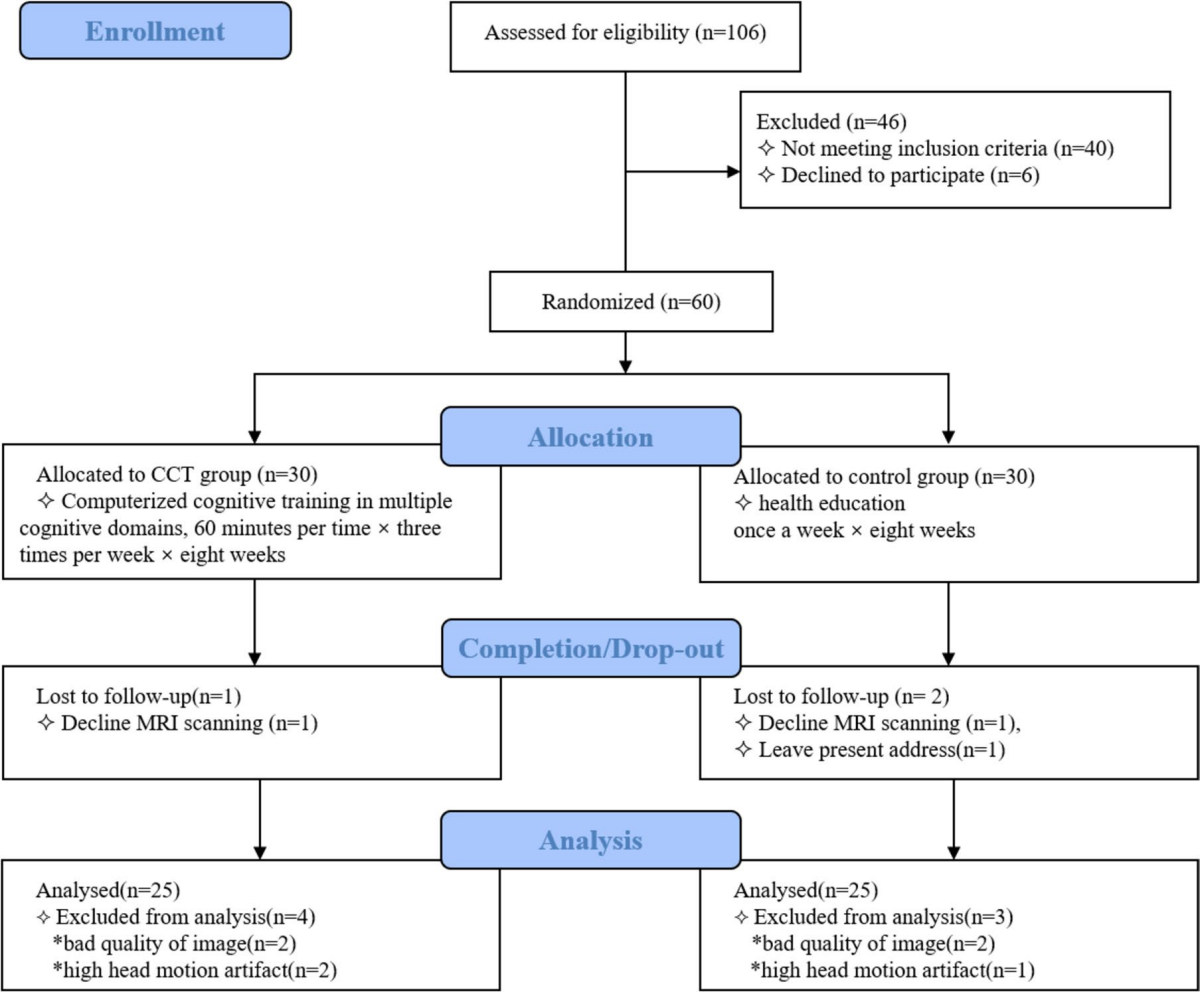
Flow diagram of the trial

Sixty MCI patients passed the screening, and 30 of them were assigned to each group (CCT and control). Three patients dropped out because they failed to complete the baseline MRI scans. Fifty-seven participants (CCT: *n* = 29; control: *n* = 28) completed the interventions and both sets of clinical assessments and brain scans. Another seven patients were excluded from our final analysis because of poor-quality MRI data or the presence of head motion artifacts—four participants based on DTI data and three participants based on resting-state image data. No adverse reactions occurred during this study. Ultimately, 50 participants were eventually included in the analysis of the results (see Fig. 1).

### Demographic characteristics and measures at baseline

There were no significant between-group differences in age, gender, years of education, body mass index, diabetes, hypertension, HAMD scores, or instrumental activities of daily living (IADL) scores (Table 1). At baseline, no significant differences were observed in the MoCA, CAVLT, Stroop, DSST, or Rey CFT scores between the CCT and control groups (*P* > 0.05) (Table 2). In addition, the differences in the SC-FC coupling in the global network and its seven component networks, the MRI head motion parameters (the maximum translation and rotation in the *X*, *Y*, and *Z* axes as well as the mean FD_Jenkinson score) were not significant (*P* > 0.05) (Supplementary Material, Tables S2, and S3).

**Table 1:** Differences in baseline demographic characteristics (mean ± SD)

	CCT group (*n* = 25)	Control group (*n* = 25)	*t*/*Z*/*χ*^2^	*P*
Age (years)^a^	67.68 ± 5.83	65.52 ± 5.55	1.341	0.186
Gender (female/male)^c^	18/7	20/5	0.439	0.508
Education (years)^a^	8.76 ± 2.95	9.84 ± 3.35	− 1.210	0.232
Body mass index (kg/m^2^)^a^	23.51 ± 3.59	22.74 ± 2.69	0.864	0.392
Diabetes (yes/no)^c^	4/21	7/18	1.049	0.306
Hypertension (yes/no)^c^	11/14	5/20	3.309	0.069
HAMD^b^	2.16 ± 2.25	1.44 ± 1.76	− 1.066	0.286
IADLs^b^	22.52 ± 0.92	22.52 ± 0.77	− 0.425	0.671

a is for continuous variables in normal distribution, examined by independent *t* test; b is for continuous variables in abnormal distribution, examined by Mann‒Whitney *U* test; and c is for categorical variables, examined by chi-square test

**Table 2:** Comparisons of between- and within-group effects on scores of neuropsychological tests

	CCT group		Control group		Group *P*	Time *P*	Group × time *P*	Within-group comparison			
	Pre	Post	Pre	Post				CCT	Control	*β*	*P*
								△(SD)	△(SD)		
MoCA	20.40 (2.42)	24.52 (2.80)	21.64 (2.27)	21.88 (3.06)	0.322	**< 0.001**	** < 0.001**	4.12 (2.26)	0.24 (1.54)	4.224	**< 0.001**
DSST	35.32 (19.19)	39.12 (18.07)	37.58 (14.41)	36.72 (14.66)	0.986	0.565	0.363	3.8 (17.38)	− 0.86 (18.49)	1.050	0.842
Stroop C-A response time	18.21 (18.90)	14.13 (11.63)	19.62 (14.76)	22.67 (17.39)	0.232	0.78	0.059	− 4.08 (17.11)	3.05 (6.96)	− 7.356	0.054
CAVLT-immediate recall	19.48 (5.39)	23.20 (5.07)	20.88 (5.13)	21.44 (5.07)	0.894	**0.001**	**0.01**	3.72 (4.63)	0.56 (3.57)	3.255	**0.008**
CAVLT-delayed recall	6.08 (3.40)	7.36 (3.74)	6.20 (2.69)	5.96 (2.48)	0.44	0.111	**0.022**	1.28 (2.30)	− 0.24 (2.22)	1.518	**0.020**
Rey CFT-recall	12.90 (7.53)	17.28 (7.67)	15.52 (6.67)	16.00 (8.72)	0.743	**0.003**	**0.015**	4.38 (6.26)	0.48 (4.47)	4.834	**0.002**

### Changes in neuropsychological test scores

There was a significant group × timepoint effect on the scores for the MoCA, CAVLT immediate and delayed recall subtests, and Rey CFT recall subtests (Table 2). No significant effects were found in Stroop or DSST scores. The CCT group showed greater changes in MoCA (*P* < 0.001), CAVLT immediate recall (*P* = 0.008), delayed recall (*P* = 0.020), and Rey CFT recall (*P* = 0.002) scores than the control group after controlling for age, gender, and years of education.

### Changes in SC-FC coupling values and their relationships with test scores

**Fig. 2:**
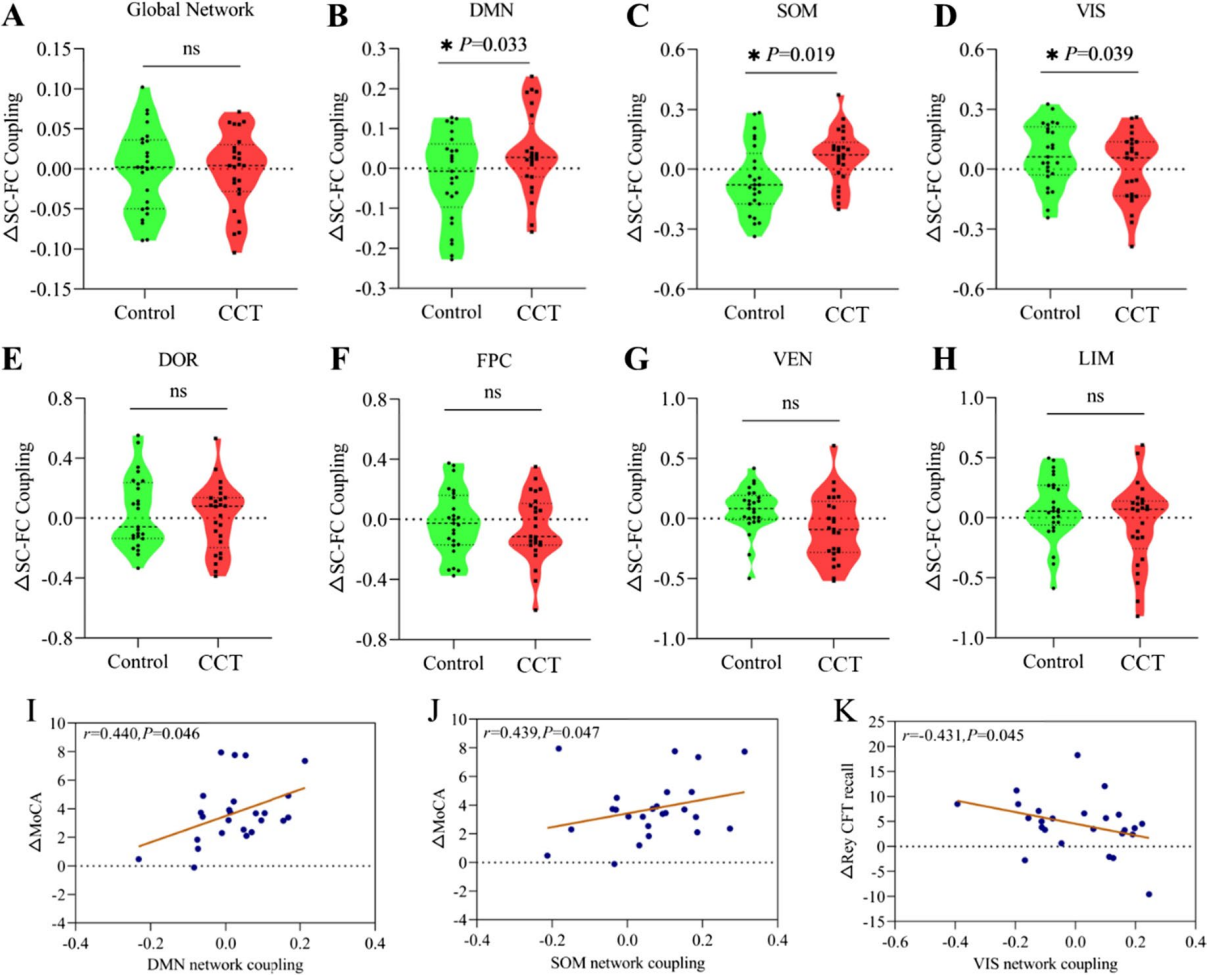
Changes in the SC-FC coupling values of neural networks in the CCT and control groups and their relationships with changes in the MoCA and Rey CFT-recall scores

Changes in the SC-FC coupling values of the DMN (*F* = 4.862, *P*-uncorrected (*P*(unc.) = 0.033) and SOM (*F* = 5.938, *P*(unc.) = 0.019) in the CCT group were larger than those in the control group, with control for age, gender, and years of education. In contrast, changes in the coupling values of VIS (*F* = 4.524, *P*(unc.) = 0.039) were smaller in the CCT group than in the control group (Fig. 2B–D). No significant between-group differences were observed in the DOR, FPC, VEN, or LIM. Increases in the SC-FC coupling values in the DMN and SOM were correlated with improvements in the CCT participants’ MoCA scores (*r* = 0.440, *P*(unc.) = 0.046; *r* = 0.439, *P*(unc.) = 0.047, respectively) (Fig. 2I, J). The SC-FC coupling values in the VIS of the CCT participants were negatively correlated with the changes in the Rey CFT recall scores (*r* =  − 0.431, *P*(unc.) = 0.045) (Fig. 2K).

### Changes in network topologies and their relationships with test scores

#### Mesoscale topology

For functional networks, the change values in the clustering coefficients of the DMN and SOM were larger in the CCT group than in the control group (*F* = 4.079, *P*(unc.) = 0.049; *F* = 14.378, *P*(unc.) < 0.001). However, there were no significant between-group differences in the network efficiencies or characteristic path lengths for these networks (*P* > 0.05). Furthermore, no significant between-group differences were observed in the clustering coefficients, network efficiencies, and characteristic path lengths in the VIS (*P* > 0.05). (Supplementary Material, Table S4).

For structural networks, no significant between-group differences were observed in the clustering coefficients, network efficiencies, or characteristic path lengths for the DMN, SOM, or VIS (*P* > 0.05) (Supplementary Material, Table S5).

#### Nodal topology

**Fig. 3:**
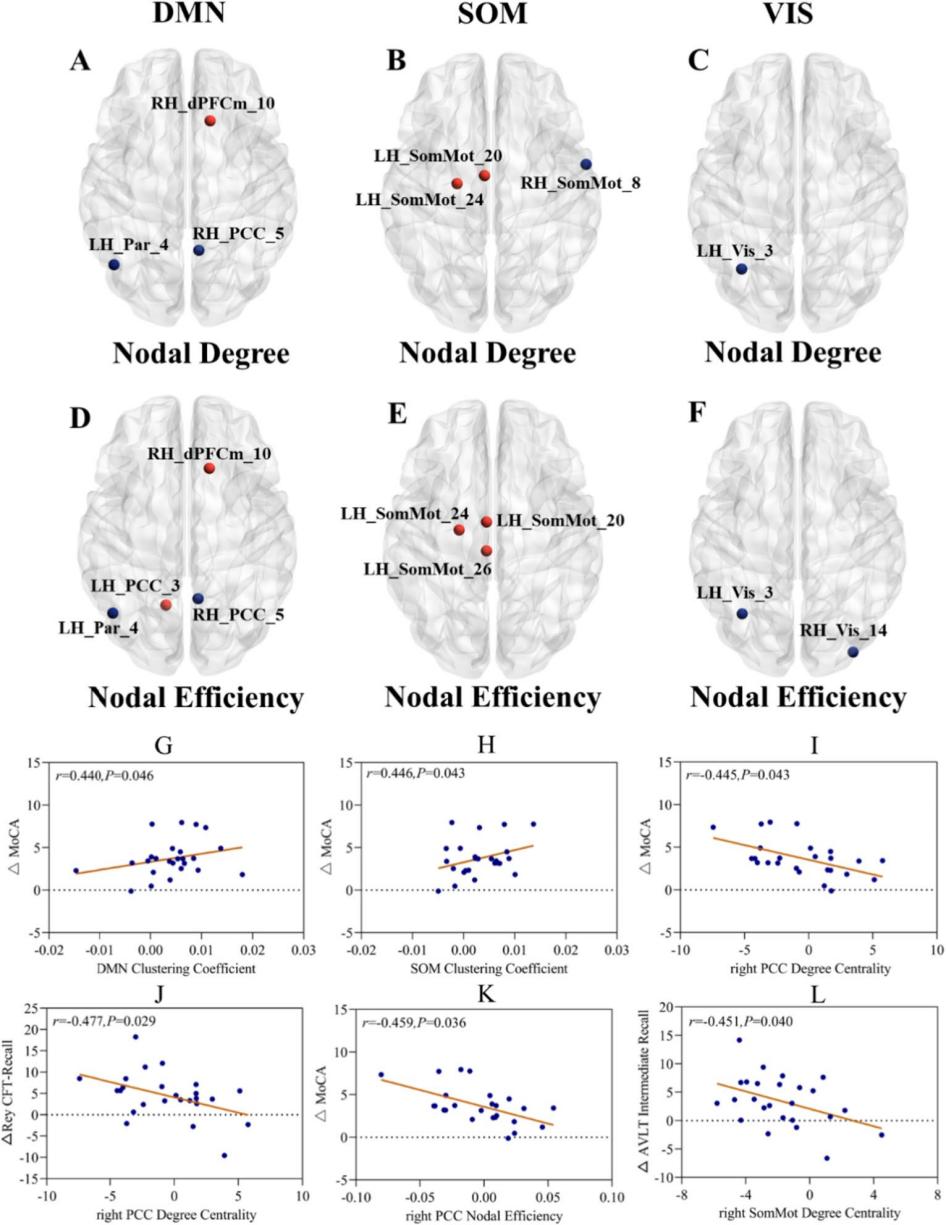
Changes in the nodal degrees and nodal efficiencies in the various functional networks between the CCT and control groups and their correlations with neuropsychological test scores. Notes: The color-coding system used above refers to the following: “red dots” denote brain regions that show significantly larger changes in nodal degrees or nodal efficiencies in the CCT group than in the control group; “blue dots” denote brain regions that show significantly smaller changes in the CCT group. Changes in the nodal degrees and nodal efficiencies reflect changes in the connectivity patterns and network topologies related to the CCT effects

Within the functional DMN, significantly larger changes in the CCT group than in the control group were observed in the nodal degree of the right dorsomedial prefrontal cortex (dPFCm) and nodal efficiencies of the right dPFCm and left posterior cingulate cortex (PCC) (*P* < 0.05; Bonferroni correction). In contrast, there were significantly smaller changes in nodal degrees and nodal efficiencies observed in the left parietal lobe (Par) and right PCC in the CCT group (*P* < 0.05; Bonferroni correction) (Fig. 3A and D).

Within the functional SOM, significantly larger changes in the CCT than in the control group were observed in the nodal degree of the left somatomotor area (SomMot), but significantly smaller changes were observed in the right SomMot (*P* < 0.05; Bonferroni correction). The nodal efficiency of the right SomMot showed significantly larger increases in the CCT group than in the control group (Fig. 3C and E).

Within the functional VIS, the CCT group displayed significantly smaller changes in the nodal degree of the left visual cortex (Vis) than the control group (*P* < 0.05; Bonferroni correction). The changes in the nodal efficiencies of the left and right VIS were also significantly smaller in the CCT group than in the control group (*P* < 0.05; Bonferroni correction) (Fig. 3B and F).

#### Relationships between neural parameters and test scores

After factoring in age, gender, and years of education, correlations were revealed between changes in the functional DMN and SOM clustering coefficients and scores of the MoCA (*r* = 0.440, *P*(unc.) = 0.046; *r* = 0.446, *P*(unc.) = 0.043, respectively) (Fig. 3G and H). Changes in the nodal degrees of the right PCC were negatively correlated with changes in the MoCA and Rey CFT recall scores (*r* =  − 0.445, *P*(unc.) = 0.043; *r* =  − 0.477, *P*(unc.) = 0.029, respectively) (Fig. 3I and J). The nodal efficiency of the right PCC was also correlated with MoCA scores (*r* =  − 0.459, *P*(unc.) = 0.036) (Fig. 3K). However, the nodal degrees of the right SomMot within the SOM were negatively correlated with the CAVLT immediate recall scores (*r* =  − 0.451, *P*(unc.) = 0.040) (Fig. 3L). If corrections for the multiple analyses were applied, all the correlations above did not reach the statistical significance threshold, i.e., *P* < 0.05/76 = 0.0006. It is, however, noteworthy that all the correlations reported were of moderate effect sizes, i.e., *r* > 0.40.

### MCID of the CCT

With the MCID thresholds, smaller groups of CCT participants showing more improvements in the clinical outcome test scores were compared with their control group counterparts. Regarding global cognitive function (MoCA test), 14 participants in the CCT group showed increases in the SC-FC coupling values of the DMN (*F* = 0.861, *P*(unc.) = 0.007) (Supplementary Material, Table S6). Regarding immediate memory function (CAVLT immediate recall test), 12 participants in the CCT group showed increases in the SC-FC coupling values of the SOM (*F* = 5.331, *P*(unc.) = 0.037), while decreases in coupling in the FPC network (*F* = 5.842, *P*(unc.) = 0.030) (Supplementary Material, Table S7). Regarding delayed memory function (CAVLT delayed recall test), 12 CCT participants showed larger changes in the SC-FC coupling values of the SOM (*F* = 5.623, *P*(unc.) = 0.030) (Supplementary Materials, Table S8). Regarding visual memory function (Rey CFT recall test), 10 CCT participants showed significant decreases in the SC-FC coupling values of the VIS network (*F* = 9.910, *P*(unc.) = 0.007) (Supplementary Material, Table S9).

## Discussion

This study aimed to provide an understanding of the positive effects of 8 weeks of CCT on global cognition and memory function in MCI patients. Significant differences between the CCT and control groups were found in global cognition and memory function and the DMN, SOM, and VIS networks. Our significant findings were that CCT resulted in increases in SC-FC coupling and global functional topography of the DMN and SOM networks but decreases in coupling of the VIS network. In addition, the CCT participants showed different patterns of changes in the nodal degree and efficiency in the left and right hemispheres. The changes in SC-FC coupling and nodal topography within different functional networks were found to be specific to the participant’s cognitive function gains. More importantly, our findings of SC-FC coupling and topography changes are consistent with the common theoretical models explaining age-related cognitive decline: the posterior–anterior shift in aging (PASA) model and the hemispheric asymmetry reduction in older adults (HAROLD) model.

In this study, we revealed that after receiving cognitive training, the participants showed increases in the SC-FC coupling and global clustering coefficients of the DMN and SOM networks, which were correlated with improvements in global cognition and memory function. In contrast, there were decreases in the SC-FC coupling of the VIS network, which were correlated with improvements in visual memory recall function. These distinct neural changes after CCT reflect the posterior-to-anterior dynamic in the neural activities described in the PASA model [46]. In the PASA model, the shift in brain activations is regarded as an age-related compensatory process from the decreases in activities in the visual and sensory cortices to the increases in activities in the prefrontal cortex. Our results suggest that CCT may enhance the neural dynamics between the posterior and anterior regions of the brain in individuals with MCI. The treatment effects of CCT would have resulted in the reorganization of the structural‒functional architecture. In addition, the changes in functional and structural network coupling and within-network nodal clustering in the VIS, DMN, and SOM networks may serve as neural markers in future aging studies. We propose that the treatment effects of CCT would have promoted reorganization of the structural‒functional architecture. Our proposition is supported by previous reports of MCI patients exhibiting abnormal changes in SC-FC coupling and topological properties in the DMN and SOM networks, which were associated with declines in cognitive function [14, 47, 48]. In particular, the disruption of the DMN network’s connections can result in the decoupling phenomenon associated with cognitive decline [49, 50]. Reductions in SC and FC in the SOM network in MCI patients were associated with decreased cognitive control function [4, 51] and deterioration of working memory, attention, and arithmetic skills [52]. Other studies revealed that cognitively impaired individuals showed abnormalities in the VIS network [53–55]. In the CCT, the 11 modules involve encoding and processing visual stimuli at different paces and various levels of tasks. Participants’ repeated exposure to visual stimuli would have modified the connectivity of the VIS network, while continuously engaging in the tasks would have strengthened the connectivity of the DMN and SOM. Visual-stimulus-induced changes in the functional connectivity of the VIS network have been previously reported in another study [56]. Chen et al. also reported that 6 months of vision-based cognitive training altered the topological properties in MCI patients, resulting in increased information processing capacity [57].

The nodal topological results suggest the possibility of CCT-related modifications of right–left lateralization in the participants. This phenomenon is consistent with the HAROLD model proposed by Cabeza et al. [58] regarding diminishing hemispheric anisotropy and increased bilateral neural activation to compensate for age-related neurodegeneration. For instance, we revealed that after training, MCI participants showed increases in the nodal efficiency of the left PCC in the functional DMN but decreases in the right PCC. The PCC has been suggested to play a major role within the DMN, subserving episodic memory retrieval [59]. Individuals with MCI also showed significant gray matter atrophy and fiber connection loss in the bilateral PCC regions [60, 61]. The finding of positive postintervention cognitive gains suggests that the CCT’s effects might have occurred via modulation of the DMN’s left–right lateralization. A previous study reported that left lateralization of the DMN was associated with memory performance [62]. Among our participants, the decreases in the nodal degree and efficiency of the right PCC were associated with improvements in general cognition and visual memory function. CCT-induced changes were also observed in the functional SOM. These changes consisted of increases in the nodal degree and efficiency of the left-sided regions but decreases in the right-sided regions. The decreases in the latter were associated with improvements in the participants’ immediate recall function. Previous studies found that the sensorimotor cortex was involved in neurocognitive processing [4, 63]. The functional SOM exhibited left lateralization, which was similar to the DMN network [64, 65].

It was evidenced that individuals could maintain good cognitive abilities and successful day-to-day functioning despite significant neuronal loss and atrophy [66]. This might hint that functional compensation occurs to maintain cognitive function even with structural atrophy. In this study, we found increases in DMN and SOM network coupling and decreases in VIS network coupling after CCT intervention. Previous studies revealed comparable findings that SC-FC coupling changes varied across different networks in the progression of MCI [67, 68]. For instance, the ReHo value and functional connectivity in the DMN were increased, while they were decreased in the central executive network and salience network. Another factor that can explain the different SC-FC change patterns revealed is structural atrophies among MCI individuals [66]. The gray matter atrophy was found to mainly be situated in the bilateral hippocampus and amygdala, extending to the medial temporal lobe and precuneus [69, 70]. The losses of white matter integrity were in the parahippocampal cingulum connecting to the hippocampus and precuneus within the DMN network [71]. These atrophies were reported to be associated with increased functional network connectivity [66]. Applicable to this study is that atrophy might have occurred in the DMN and SOM networks rather than in the VIS networks. The DMN network is in the connectivity between the mPFC and inferior parietal lobe, while the SOM network is in the supplementary motor areas and superior parietal lobe [67, 68]. The potential structural atrophies would have weakened the structural connectivity and hence the SC-FC relationships in the DMN and SOM networks. The increases in SC-FC coupling after CCT would have acted on the atrophic changes due to the MCI pathologies in the DMN and SOM networks. In contrast, the structural atrophy in the VIS network, if any, would have been less significant than that in the DMN and SOM networks. Different patterns of SC-FC coupling yielded for the VIS network can be expected. Nevertheless, as structural atrophies were not measured in this study, our speculations on the DMN and SOM versus the VIS network need to be further verified in future studies.

The CCT-induced changes in SC-FC coupling and nodal topography indicate the neuroplastic characteristics of the MCI participants. The plastic changes covered the functional DMN, SOM, and VIS. Notably, these neural changes were found to correspond to specific improvements in cognitive function. These results further illustrate the scaffolding theory of aging and cognition–revised (STAC-r) proposed by Reuter-Lorenz PA et al. [72]. The STAC-r states that older adults utilize compensatory strategies, such as reconfiguring their cognitive and neural resources, to cope with age-related cognitive decline. The improved cognitive function among the participants suggests the possibility that CCT might have initiated better-compensated MCI-related brain function changes. The contents of the CCT and the design of this study, i.e., the lack of a healthy older adult group, did not allow us to further explore the dose–response relationship at the module level and, hence, the proposed reversal of age-related compensation. Future studies should use training modules designed with discrete task content and manipulate both the content and the dosage of training to address our proposition.

## Limitations

The study has several limitations. First, the sample sizes were relatively small due to the strict selection criteria set for the study. The small sample sizes, in particular, were among those showing test score changes above the MCID thresholds for the between-group comparisons. This could have lowered the power of the analyses, causing an underestimation of the effects of the CCT on SC-FC coupling and nodal topography. Second, corrections for the multiple comparisons and correlational analyses were not applied to selected secondary outcome variables. Readers are reminded to take caution when interpreting the findings on brain network parameter changes and their relationships with improvements in CCT-related cognitive functions. Future studies should consider setting specific a priori hypotheses on testing the brain network to behavioral relationships related to CCT. Third, individuals with MCI are heterogeneous. The inclusion criteria do not stipulate the disease process behind cognitive decline, such as early neurodegenerative disease or Alzheimer’s disease. The readers are reminded to be cautious with generalizing the results of the CCT effects to MCI individuals with specific pathologies. Fourth, because of the lack of a follow-up design, the long-lasting effect of the CCT remains to be explored in future studies. Another limitation is the possible learning effects developed among the participants due to the use of the same neuropsychological tests as the outcome measures. Although the learning effects, if any, would have been accounted for by the experimental-control group design, future studies should consider conducting pre- and post-training testing with instruments having parallel or alternate forms.

## Conclusion

Eight weeks of CCT appear to induce changes in the structural‒functional coupling and nodal topography of the DMN, SOM, and VIS. Changes in the different networks were found to be associated with improvements in general cognitive function and memory function in individuals with MCI. Furthermore, our findings illustrate that CCT might be beneficial for modulating network properties and improving cognitive functions in individuals with MCI.

### Supplementary Information

**Additional file 1: Figure S1.** The workflow of this study. **Table S1.** Descriptions of the 11 modules of the computerized cognitive training (CCT) program. **Table S2.** for the comparison of group differences in the baseline SC-FC couplings. **Table S3.** For the comparison of group differences in the head motion parameters. **Table S4.** For the comparison of group differences in the functional global topology. **Table S5.** For the comparison of group differences in the structural global topology. **Table S6.** For the comparison of differences in network coupling selected according to MoCA scores. **Table S7.** For the comparison of differences in network coupling that were selected according to scores of the CAVLT immediate recall test. **Table S8.** For the comparison of differences in network coupling that were selected according to scores of the CAVLT delayed recall test. **Table S9.** For the comparison of differences in network coupling that were selected according to scores of the Rey CFT recall test.

## Data Availability

The datasets used during the current study are available from the corresponding author upon reasonable request.

## Abbreviations

CAVLT: Chinese Auditory Verbal Learning Test
BMI: Body mass index
CCT: Computerized cognitive training
DMN: Default mode network
DOR: Dorsal attention network
dPFCm: Dorsal medial prefrontal cortex
DTI: Diffusion tensor image
FOV: Field of view
FPC: Frontoparietal control network
GLM: General linear model
HAMD: Hamilton Depression Scale
IADLs: Instrumental activity of daily living scale
LIM: Limbic network
MCI: Mild cognitive impairment
MoCA: Montreal Cognitive Assessment
PCC: Posterior cingulate cortex
ROCFT: Rey-Osterrieth Complex Figure Test
rs-fMRI: Resting state-functional magnetic resonance image
SOM: Somatomotor network
TE: Echo time
TR: Repetition time
VEN: Ventral attention network
VIS: Visual network
