# Supplementary material for: Effects of computerized cognitive training on structure‒function coupling and topology of multiple brain networks in people with mild cognitive impairment: a randomized controlled trial
Source: Alzheimers Res Ther. 2023 Sep 23;15:158. doi: 10.1186/s13195-023-01292-9 (PMC10517473; doi:10.1186/s13195-023-01292-9)
Supplement: Supplementary file 1 — Additional file 1: Figure S1. The workflow of this study. Table S1. Descriptions of the 11 modules of the computerized cognitive training (CCT) program. Table S2. for the comparison of group differences in the baseline SC-FC couplings. Table S3. For the comparison of group differences in the head motion parameters. Table S4. For the comparison of group differences in the functional global topology. Table S5. For the comparison of group differences in the structural global topology. Table S6. For the comparison of differences in network coupling selected according to MoCA scores. Table S7. For the comparison of differences in network coupling that were selected according to scores of the CAVLT immediate recall test. Table S8. For the comparison of differences in network coupling that were selected according to scores of the CAVLT delayed recall test. Table S9. For the comparison of differences in network coupling that were selected according to scores of the Rey CFT recall test. [file 13195_2023_1292_MOESM1_ESM.docx]

**Supplementary Materials**

**Methods**

*Structural-functional connectivity (SC-FC)*. Preprocessed diffusion image was co-registered to the T1 image and then non-linearly transformed to the Montreal Neurological Institute (MNI) space using the MNI_152_T1 brain template. Then, we inversed the derived transformation parameters and applied the ROI template to wrap the Schaefer-300 atlas from the MNI space to the diffusion native space. After this step, we conducted deterministic fiber tracking using PANDA to derive the FA matrix in two steps: the edges between two nodes were considered to be structural connectivity when the FA value of deterministic fiber tracts was between 0.2 and 1.0. Then structural networks were constructed by 300×300 matrices. Preprocessed resting-state fMRI data were segmented into 300 nodes to construct the functional connectivity matrix. The time series for each brain region was derived by computing the average for the fMRI time series over all voxels. Next, we extracted the average time courses of all ROIs to calculate the Pearson correlation coefficients for each pair of regions. Finally, we constructed the undirected and binary functional connectivity networks.

*SC-FC correlation (coupling)*. Pearson's correlation coefficients were computed between the structural connectivity matrix and functional connectivity matrix. We only correlated the non-zero edges in the structural connectivity with the positive functional connectivity. The correlation coefficients for each participant represented the SF coupling.

**Figure S1.** The workflow of this study.


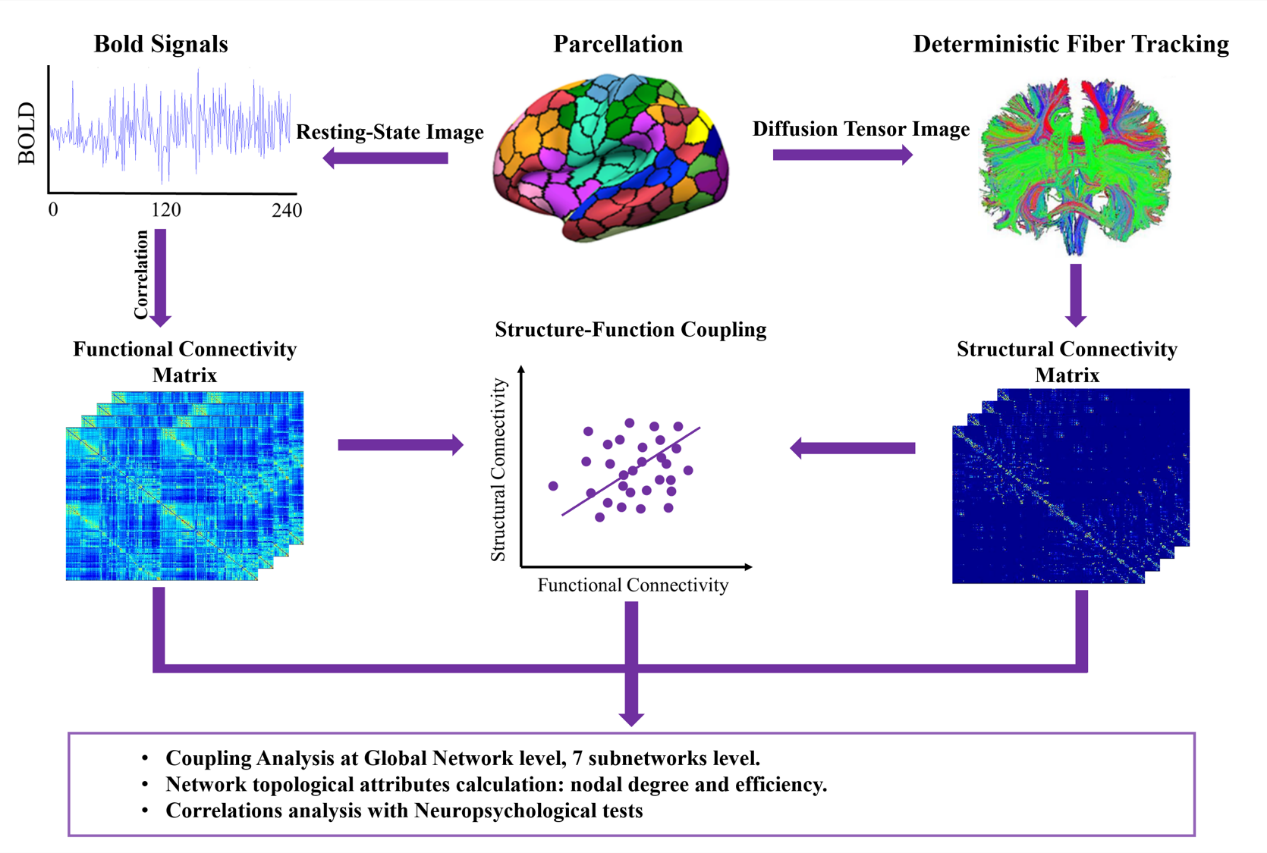


The resting-state image and diffusion tensor image were applied to construct the functional/structural connectivity matrix according to the Schaefer-300 parcellation atlas. There we conducted the Pearson’s correlation between functional connectivity and structural connectivity to obtain the structure-function coupling. In this study, we probed the coupling at the network level (including the global network and seven subnetworks defined by Yeo et al. atlas). For the topology analysis, we detected subtle changes using the nodal-wise topological properties in the subnetworks.

**Supplementary Table**

**Table S1. Descriptions of the 11 modules of the computerized cognitive training (CCT) program.**

| **No** | **Names** | **Descriptions** | **Difficulty parameters** |
| --- | --- | --- | --- |
| 1 | Warm Up | Encode objects and respond to targets | Nil |
| 2 | Visuospatial Function | Discriminate a target object from the other distractor, and click as quickly as possible on the target object | Similarity of the sizes, shapes, colors, and/or spatial orientation of the target and distractor objects |
| 3 | Auditory Discrimination | Compare two pieces of short soundtracks, and indicate as quickly as possible whether they are the same | Similarity of the tonal and melodic features of the two soundtracks |
| 4 | Visual Search | Compare target from distractor objects, and quickly locate and click as quickly as possible on the target object | Similarity of the sizes and shapes between the target and distractor objects, and increase in the number of the distractor objects |
| 5 | Attention – Sustained | Compare the stream of target and distractor object presentations, while the targets are oddball in appearance, and click as quickly as possible on the target objects | Increase the duration of the stream of the object presentations; and reduce presentation times of each object presentation |
| 6 | Attention – Selection | Observe and maintain a target object in mind, then select the target from distractor objects, and click as quickly as possible on the target object; repeat the same task process | Similarity of the outlooks between the target and distractor objects, and increase the duration of the stimulus presentation |
| 7 | Attention – Divided | Observe and maintain a target object in mind, then select targets from distractor objects presented simultaneously at different paces | Similarity of the outlooks between the target and distractor objects, and reduce the time gaps between stimulus presentations |
| 8 | Working Memory | Compare the stimulus that has just appeared with one previous designated stimulus (nth-back), and press the key on the keyboard to indicate a match between the two stimuli | Number of the nth-back from one to three, and the similarity between the target and distractor stimuli |
| 9 | Planning | Visualize the path of the maze from “start” to “exit”, and draw a continuous path on the maze without correction | Size of the maze, and complexity and number of alternative paths in the maze |
| 10 | Response Inhibition  （Go/No Go） | Learn the object rule for “go” or “no-go” response, and press the corresponding keys on the keyboard in response to the object presentations | Gap time between the stimulus presentation, and the similarity of the outlooks between the target and distractor stimuli |
| 11 | Mental Rotation | Extract figure features of the target object, and select the object in varying orientations and with matched features by pressing the key on the keyboard | Angle of rotation of the object, number of the distractor objects, and similarity of object features among objects |

**Table S2 for the comparison of group differences in the baseline SC-FC couplings**

| Table S2 Comparison of baseline SC-FC couplings between groups (mean±SD) | | | | |
| --- | --- | --- | --- | --- |
|  | CCT group (n=25) | Control group(n=25) | t/Z | *P* |
| Global | -0.27081±0.05588 | -0.28248±0.05291 | 0.758 ^a^ | 0.452 |
| DMN | -0.24555±0.10607 | -0.26309±0.12324 | 0.539 ^a^ | 0.592 |
| DOR | -0.15627±0.14648 | -0.20150±0.17638 | 0.986 ^a^ | 0.329 |
| FPC | -0.02204±0.21389 | -0.04863±0.20031 | 0.454 ^a^ | 0.652 |
| LIM | -0.14789±0.25149 | -0.24893±0.24510 | 1.439 ^a^ | 0.157 |
| SOM | -0.17505±0.12641 | -0.13470±0.14469 | -1.232 ^b^ | 0.218 |
| VEN | -0.18016±0.19975 | -0.24196±0.21867 | 1.043 ^a^ | 0.302 |
| VIS | -0.10134±0.13570 | -0.14555±0.11668 | 1.235 ^a^ | 0.223 |
| Notes: a for the t score of the independent t-test; b for the Z score of the Mann-Whitney U test. | | | | |

**Table S3 for the comparison of group differences in the head motion parameters**

| Table S3 Comparison of head motion parameters between groups at baseline and post-intervention (mean±SD) | | | | | | | |
| --- | --- | --- | --- | --- | --- | --- | --- |
|  | Baseline | | |  | Post-intervention | | |
|  | CCT group (n=25) | Control group (n=25) | *P* |  | CCT group (n=25) | Control group (n=25) | *P* |
| max Translation in X axis (mm) | 0.26196±0.21564 | 0.28263±0.18191 | 0.503 |  | 0.34388±0.17849 | 0.33722±0.20786 | 0.554 |
| max Translation in Y axis (mm) | 0.29709±0.19194 | 0.29074±0.24494 | 0.443 |  | 0.24443±0.10758 | 0.29716±0.18211 | 0.432 |
| max Translation in Z axis (mm) | 0.78514±0.58008 | 0.64377±0.36185 | 0.662 |  | 0.73584±0.53402 | 0.69450±0.35852 | 0.764 |
| max Rotation in X axis (degree) | 0.64328±0.48249 | 0.65271±0.40038 | 0.541 |  | 0.66911±0.50866 | 0.79984±0.57997 | 0.443 |
| max Rotation in Y axis (degree) | 0.37976±0.32189 | 0.48990±0.48699 | 0.337 |  | 0.47785±0.29369 | 0.44382±0.31855 | 0.594 |
| max Rotation in Z axis (degree) | 0.37541±0.34631 | 0.40677±0.38751 | 0.823 |  | 0.39884±0.37085 | 0.35298±0.21382 | 0.854 |
| mean FD_Jenkinson | 0.09847±0.05343 | 0.08386±0.04321 | 0.410 |  | 0.12663±0.09925 | 0.08490±0.04248 | 0.160 |

**Table S4 for the comparison of group differences in the functional global topology**

| Table S4 Comparison of functional global topology between groups (mean±SD) | | | | |
| --- | --- | --- | --- | --- |
|  | CCT group (n=25) | Control group(n=25) | F | *P* |
| **DMN network** |  |  |  |  |
| Network Efficiency | 0.00392±0.01145 | -0.00129±0.01543 | 1.277 | 0.264 |
| Clustering Coefficient | 0.00407±0.00743 | -0.00357±0.01342 | 6.172 | **0.017** |
| Characteristic Path Length | -0.02276±0.09279 | 0.02294±0.10430 | 2.587 | 0.115 |
| **SOM network** |  |  |  |  |
| Network Efficiency | 0.00725±0.01209 | 0.00038±0.01248 | 3.719 | 0.060 |
| Clustering Coefficient | 0.00305±0.00668 | -0.00226±0.01229 | 7.502 | **0.009** |
| Characteristic Path Length | -0.02421±0.07871 | 0.02579±0.10090 | 3.658 | 0.062 |
| **VIS network** |  |  |  |  |
| Network Efficiency | 0.00722±0.03314 | -0.00112±0.01918 | 1.743 | 0.193 |
| Clustering Coefficient | 0.00225±0.01921 | -0.00011±0.01392 | 0.314 | 0.578 |
| Characteristic Path Length | -0.03899±0.16496 | -0.00302±0.12762 | 1.557 | 0.219 |
| Notes: Controlling age, gender, and years of education | | | | |

**Table S5 for the comparison of group differences in the structural global topology**

| Table S5 Comparison of structural global topology between groups (mean±SD) | | | | |
| --- | --- | --- | --- | --- |
|  | CCT group (n=25) | Control group(n=25) | F | *P* |
| **DMN network** |  |  |  |  |
| Network Efficiency | 0.00644±0.01719 | -0.00388±0.01139 | 3.957 | 0.053 |
| Clustering Coefficient | 0.01297±0.03509 | -0.00386±0.02915 | 2.124 | 0.152 |
| Characteristic Path Length | -0.01841±0.88825 | 0.11151±0.49740 | 0.001 | 0.974 |
| **SOM network** |  |  |  |  |
| Network Efficiency | 0.00918±0.02158 | 0.00121±0.02273 | 0.667 | 0.418 |
| Clustering Coefficient | 0.00987±0.03572 | 0.00443±0.03703 | 0.154 | 0.697 |
| Characteristic Path Length | -0.52004±1.08992 | 0.00395±0.73341 | 2.417 | 0.127 |
| **VIS network** |  |  |  |  |
| Network Efficiency | 0.00036±0.00821 | -0.00279±0.01208 | 0.895 | 0.349 |
| Clustering Coefficient | 0.00535±0.01619 | 0.00018±0.02175 | 0.160 | 0.691 |
| Characteristic Path Length | 0.00453±0.27237 | 0.01836±0.09103 | 0.076 | 0.784 |
| Notes: Controlling age, gender, and years of education | | | | |

**Table S6 for the comparison of differences in network coupling selected according to MoCA scores**

| Table S6 Differences of network coupling selected according to MoCA | | | | |
| --- | --- | --- | --- | --- |
|  | CCT group (n=14) | Control group (n=14) | F | *P* |
| DMN | 0.06254±0.07676 | -0.04684±0.11341 | 9.118 | **0.006** |
| DOR | 0.08310±0.20375 | -0.02332±0.23427 | 1.953 | 0.176 |
| FPC | -0.00480±0.20568 | 0.05838±0.22835 | 0.882 | 0.358 |
| LIM | -0.04685±0.36098 | 0.11274±0.22190 | 1.376 | 0.253 |
| SOM | 0.03975±0.11186 | -0.06318±0.17920 | 2.857 | 0.105 |
| VEN | -0.04880±0.28186 | 0.13692±0.13320 | 3.408 | 0.078 |
| VIS | 0.03247±0.16236 | 0.08753±0.14463 | 1.521 | 0.230 |
| Notes: Controlling age, gender, and years of education | | | | |

**Table S7 for the comparison of differences in network coupling that were selected according to scores of the CAVLT immediate recall test.**

| Table S7 Differences of network coupling selected according to CAVLT immediate recall | | | | |
| --- | --- | --- | --- | --- |
|  | CCT group (n=12) | Control group (n=12) | F | *P* |
| DMN | 0.02670±0.13074 | -0.00635±0.10627 | 1.347 | 0.265 |
| DOR | 0.05565±0.25019 | -0.02284±0.1905 | 0.118 | 0.737 |
| FPC | -0.06419±0.20949 | 0.01258±0.18918 | 5.842 | **0.030** |
| LIM | 0.01341±0.29823 | 0.02911±0.27344 | 0.012 | 0.914 |
| SOM | 0.08412±0.13880 | -0.00164±0.17355 | 5.331 | **0.037** |
| VEN | -0.08395±0.34113 | 0.11376±0.18085 | 4.398 | 0.055 |
| VIS | 0.02390±0.17301 | 0.08304±0.17197 | 1.594 | 0.227 |
| Notes: Controlling age, gender, and years of education | | | | |

**Table S8 for the comparison of differences in network coupling that were selected according to scores of the CAVLT delayed recall test.**

| Table S8 Differences of network coupling selected according to CAVLT delayed recall | | | | |
| --- | --- | --- | --- | --- |
|  | CCT group (n=12) | Control group (n=12) | F | *P* |
| DMN | -0.01993±0.11965 | -0.02618±0.10419 | 0.238 | 0.632 |
| DOR | 0.09995±0.21076 | -0.00429±0.23543 | 2.587 | 0.126 |
| FPC | 0.01180±0.16685 | -0.01831±0.21225 | 0.510 | 0.485 |
| LIM | -0.01798±0.37842 | 0.07160±0.29935 | 0.394 | 0.539 |
| SOM | 0.08110±0.13188 | -0.03136±0.17345 | 5.623 | **0.030** |
| VEN | -0.02573±0.28256 | 0.09452±0.19062 | 0.730 | 0.405 |
| VIS | 0.00223±0.20216 | 0.06346±0.16564 | 1.120 | 0.305 |
| Notes: Controlling age, gender, and years of education | | | | |

**Table S9 for the comparison of differences in network coupling that were selected according to scores of the Rey CFT recall test.**

| Table S9 Differences of network coupling selected according to Rey CFT recall | | | | |
| --- | --- | --- | --- | --- |
|  | CCT group (n=10) | Control group (n=10) | F | *P* |
| DMN | 0.05352±0.11024 | -0.02603±0.13137 | 1.212 | 0.289 |
| DOR | 0.08624±0.21682 | 0.05147±0.22082 | 0.566 | 0.465 |
| FPC | -0.06044±0.28662 | 0.05335±0.19581 | 0.726 | 0.409 |
| LIM | -0.14706±0.34401 | -0.01077±0.27916 | 0.320 | 0.581 |
| SOM | 0.00634±0.13516 | -0.11062±0.16594 | 3.598 | 0.079 |
| VEN | 0.01231±0.29862 | 0.03366±0.08678 | 0.182 | 0.676 |
| VIS | -0.08090±0.13757 | 0.13082±0.14044 | 9.910 | **0.007** |
| Notes: Controlling age, gender, and years of education | | | | |
